# Perceived synchrony for realistic and dynamic audiovisual events

**DOI:** 10.3389/fpsyg.2015.00736

**Published:** 2015-06-02

**Authors:** Ragnhild Eg, Dawn M. Behne

**Affiliations:** ^1^Simula Research LaboratoryOslo, Norway; ^2^Department of Psychology, Norwegian University of Science and TechnologyTrondheim, Norway

**Keywords:** multisensory perception, audiovisual synchrony, temporal integration, complex stimuli, visual distortion

## Abstract

In well-controlled laboratory experiments, researchers have found that humans can perceive delays between auditory and visual signals as short as 20 ms. Conversely, other experiments have shown that humans can tolerate audiovisual asynchrony that exceeds 200 ms. This seeming contradiction in human temporal sensitivity can be attributed to a number of factors such as experimental approaches and precedence of the asynchronous signals, along with the nature, duration, location, complexity and repetitiveness of the audiovisual stimuli, and even individual differences. In order to better understand how temporal integration of audiovisual events occurs in the real world, we need to close the gap between the experimental setting and the complex setting of everyday life. With this work, we aimed to contribute one brick to the bridge that will close this gap. We compared perceived synchrony for long-running and eventful audiovisual sequences to shorter sequences that contain a single audiovisual event, for three types of content: action, music, and speech. The resulting windows of temporal integration showed that participants were better at detecting asynchrony for the longer stimuli, possibly because the long-running sequences contain multiple corresponding events that offer audiovisual timing cues. Moreover, the points of subjective simultaneity differ between content types, suggesting that the nature of a visual scene could influence the temporal perception of events. An expected outcome from this type of experiment was the rich variation among participants' distributions and the derived points of subjective simultaneity. Hence, the designs of similar experiments call for more participants than traditional psychophysical studies. Heeding this caution, we conclude that existing theories on multisensory perception are ready to be tested on more natural and representative stimuli.

## 1. Introduction

Current knowledge about multisensory processes and percepts stems from a long line of well-designed and well-controlled research studies (McGrath and Summerfield, 1985; Lewkowicz, 1996; Stone et al., 2001; Zampini et al., 2003; Fujisaki and Nishida, 2005; Zampini et al., 2005). Common for these studies are isolated stimuli, such as tones, lights and moving discs, isolated surroundings, typically sound- and light-deprived environments, and isolated interactions, where participants simply attend and respond. These controlled approaches are essential when exploring fundamental perceptual processes. However, they do not represent the everyday world, where the senses are continuously conveying diverse multisensory inputs from the surroundings. In turn, generalizing from one situation to the next can be problematic due to differences in dynamicity, complexity, repetitiveness, and irrelevant elements. Fortunately, this body of controlled experimental research provides a solid foundation on which to build more applied investigations. We present a study motivated by the widespread use of isolated and simple experimental stimuli in audiovisual research, where we take one step toward more ecologically valid designs. In this work, we consider the temporal integration of three different audiovisual events: chess, drumming, and speech. We compare a short and a long version of each of these events, keeping focus on the potential variations in temporal integration as dependent on the eventfulness of the audiovisual stimuli. Furthermore, we control availability of visual information through reduced spatial granularity using Gaussian blur filters, allowing for an investigation into the importance of fine-grained visual details in the temporal alignment of audiovisual signals.

Perceived audiovisual synchrony varies greatly across different contexts, such as content type (Vatakis and Spence, 2010; Stevenson and Wallace, 2013), content complexity (Fujisaki and Nishida, 2005; Arrighi et al., 2006), and experimental set-ups (van de Par et al., 2002; van Eijk et al., 2008; Vatakis et al., 2008b). Individuals also differ in their sensitivity to this type of temporal discrepancy (Fouriezos et al., 2007). Findings even suggest that those experienced in highly time-dependent activities, such as musicicians (Lee and Noppeney, 2011) and gamers (Donohue et al., 2010), are more adept at making judgements on audiovisual synchrony. Moreover, the detection of asynchrony is not based on absolute thresholds; it takes the form of a dynamic range of intervals that are shaped by interacting events (Roseboom et al., 2009). Human perception can maintain coherence by allowing fairly large temporal offsets between an auditory and a visual signal to go unnoticed. However, the extent of unnoticeable asynchrony depends on which modality precedes the other. The perceptual system is more sensitive to detecting asynchrony when the auditory signals arrive before the visual signals (audio lead asynchrony) than to visual signals that arrive first (audio lag asynchrony) (Lewkowicz, 1996; Grant et al., 2003).

When it comes to content, the nature and the dynamics of the audiovisual event will likely influence the perceptual sensitivity to asynchrony. For instance, an experiment comparing judgements of synchrony between still and moving audiovisual stimuli established an 111 ms audio lead detection threshold for a static disc paired with a noise-burst, significantly different from the 79 ms threshold for a moving disc paired with a descending tone (van Eijk et al., 2008). Moreover, investigations into the temporal integration of drumming movements and corresponding drum-beats have found that the perception of synchrony holds longer for slow tempos, compared to faster tempos (Arrighi et al., 2006; Petrini et al., 2009). This implies that asynchrony is more easily detected for faster drum-beats, a notion that is supported by wider windows of temporal integration for the slow beat (for example, ≈200 ms for 60 beats-per-minute vs. ≈150 ms for 120 beats-per-minute, Petrini et al., 2009). The same point-light drummer stimuli were also contrasted with a recording of a man speaking a single word, with results revealing a greater tolerance to asynchrony in speech (Love et al., 2013). These findings demonstrate a propensity for the perceptual system to maintain temporal coherence longer for still vs. moving stimuli, as well as for slow vs. rapid series, and dynamic vs. repetitive events.

The outlined research studies into the influence of movement and dynamics on audiovisual describe controlled experiments that apply fairly simple visual stimuli and isolated auditory signals. In the physical world, and in the now ubiquitous digital world, events are not so uncomplicated and undisturbed. Possibly, the complexity and lack of control that follow the use of more realistic stimuli are part of the reason why there are so few studies that look into this topic. Fortunately, some have endeavored to explore how humans perceive synchrony for more complex audiovisual presentations. The landmark work of Dixon and Spitz (1980) showed that the detection of gradually introduced asynchrony occurred at smaller displacements for an action-oriented video than for a speech video. In fact, their video portraying a hammer hitting a peg resulted in a temporal window where asynchrony was not detected between about 75 ms audio lead and 188 ms audio lag. When the video showed a narrator, the temporal window expanded to about 131 ms audio lead and 258 ms audio lag. A consistent temporal sensitivity to asynchrony in action-oriented sequences was evident in comparison to both speech and music-related content (Vatakis and Spence, 2006). In the temporal order judgement experiment, the action also centered around the impact between a tool and an object, while the speech consisted of spoken sentences and the music contained notes played on a piano or a guitar. Moreover, the results from the study by Vatakis and Spence (2006) illustrated that subjective temporal perception can shift depending on the presented content. True temporal coincidence between an auditory and a visual signal rarely corresponds to individual reports of synchrony, and in their study, the speech and piano music stimuli showed subjective synchrony when audio preceded video, whereas the subjective mid-points for action and guitar music indicated that the video should come first. In another study with similar comparisons between simple stimuli and more complex stimuli portraying either speech or tool actions, the temporal perception of audiovisual speech yielded wider and more symmetrical distributions of perceived synchrony, irrespective of the applied experimental task (Stevenson and Wallace, 2013). In line with Dixon and Spitz (1980) and Vatakis and Spence (2006), the findings of Stevenson and Wallace (2013) imply that audiovisual asynchrony is more difficult to the detect for speech than for action-oriented sequences with more pedictable moments of impact.

Temporal integration varies within content categories, as well as between. When it comes to speech for instance, temporal misalignments are detected at shorter intervals for the more visually salient bilabial syllables, compared the less visible velar and alveolar syllables, when the auditory signal lags behind the visual (Vatakis et al., 2012). Relatedly, asynchronous speech signals can influence the identification of speech tokens (Massaro et al., 1996); interestingly though, in another experiment this effect was only evident for two-syllable stimuli and not for single-syllable stimuli (Smeele, 1994). Although the latter may be a better approximation to sporadic speech, most everyday conversations convey more than one or two syllables at the time. An investigation into the importance of media synchronization used a male speaker in a newsroom setting to establish a range of acceptable audiovisual delays (Steinmetz, 1996). The study included three different proximity conditions, a head view, a shoulder view, and a torso view, along with five language conditions and two additional content conditions, a violonist in concert and hammer hitting a nail. Unfortunately, the author does not provide details on the other languages or contents, beyond the mention of similar results. However, he states that the proximity to the English-speaking narrator does affect the detection of synchronization errors, the closer the view of the speaker, the more obvious is the asynchrony. So far, research using more applied, dynamic, or representative stimuli is limited in this domain, thus we set out to contribute more findings on potential distinctions in temporal perception between different audiovisual events.

Past research has established that the integration of auditory and visual information depends on several factors beyond timing. A prominent example from audiovisual speech perception relates to the spatial location of a seen and heard speaker (Jack and Thurlow, 1973; Bishop and Miller, 2011). On the neuronal level, the combination of multisensory signals relating to a single event is strengthened by information that converges across several dimensions (Stanford and Stein, 2007). A study into the neural processing of ecologically valid stimuli, such as speech syllables, handclapping, and spoon-tapping, showed that certain neural mechanisms involved in speeding up the identification of bimodal speech can also facilitate the processing of other audiovisual events (Stekelenburg and Vroomen, 2007). However, this facilitatory mechanism was absent when the visual modality provided no anticipatory visual event, which was the case for two additional stimuli, a paper-tearing and a sawing action. According to the authors, the lack of a crossmodal effect for these stimuli implies that is not the content itself, nor its nature, that contributed to the facilitated integration; instead, they believe it to be connected to the temporal relation between corresponding auditory and visual events. The notion that the predictability of events can come into play in temporal sensory interactions is further by findings from a creative experimental set-up by Levitin et al. (2000). They had performers and observers make simultaneity judgements on the same motoric event. The performers would use a wand to hit a table in front of them, while both the performer and the observer received the corresponding sound through headphones, early, late or in real-time. The performers, producing the sound, were quicker to notice the temporal misalignent compared to the observers. This higher temporal sensitivity to self-produced over observed actions may be connected to the subjective awareness of an anticipated moment of impact, which in turn would suggest that the predictability of temporal events influence their perception.

Temporal coincidence between sensory signals is undoubtedly important to their integration, but information specific to the content of an event is still likely to contribute to perceived correspondence between modalities. Semantic content and context remain important to the integration of auditory and visual speech, on the neuronal level (Doehrmann and Naumer, 2008), and for perceptual processes related to comprehension (Windmann, 2004) and temporal order (Vatakis et al., 2008a). Furthermore, blurred or otherwise distorted visual features tend to increase the perceptual reliance on the auditory speech signal (MacDonald et al., 2000; Thomas and Jordan, 2002; Munhall et al., 2004; Eg and Behne, 2009). Yet, interestingly, even very sparse visual information can benefit the perceptual system in identifying speech signals (Rosenblum et al., 1996; Bernstein et al., 2004). Although the quality of the visual signal affects its intelligibility in speech perception, this does not necessarily imply that the temporal integration of the auditory and visual modalities is affected by the loss of sensory information. Some findings suggest that the intensity of a light stimulus can influence its temporal processing (Roufs, 1963; Bachmann et al., 2004), yet the impact of visual quality of information on audiovisual synchrony perception is by and large an area yet to explored.

We commenced this work with a project that explored the role of both auditory and visual signal distortion on the temporal integration of the two modalities (Eg et al., 2015). In this study we focus on event complexity, which in the context of the study relates to the natural occurence of irrelevant elements and events alongside the event in focus. Our premise states that the extent to which the human perceptual system can preserve the subjective perception of audiovisual synchrony for different content depends on the relative ease of discerning and aligning the auditory and visual temporal events. In other words, we surmise that cues for the temporal alignment of modalities are tied to the clarity of the correspondence between, in this case, auditory and visual events, and we expect this to differ across content types. In addition, we assume that the multiple audiovisual events that will take place in long-running excerpts provide several temporal cues, whereas short or isolated events may only offer one audiovisual correspondence. Consequently, the repeated exposure to misaligned audiovisual events could make it easier to detect asynchrony for continuous events.

In order to test our premise, we conducted an experiment with a short and a long version of three audiovisual stimuli that contain action, music, and speech content. For our action-oriented sequence, we selected a movie scene with a game of chess taking place. The long version includes five full chess moves, while the short excerpt shows a single chess piece being placed down on the board. For the music content, we used a drumming video that starts out with a sequence of events with clear audiovisual correspondence, where the drummer is hitting his drumsticks together above his head. The short version of this content portrays a single move of the drumsticks clicking together. Finally, a news broadcast served as a representation of the dynamic speech typical of everyday language, and the recording of the spoken syllable /ba/ offered a controlled, singular speech token. By choosing a bilabial stop consonant, we ensured that both the auditory and visual speech tokens would have high perceptual salience (Kent, 1997); additionally, these salient speech sounds have been found to yield greater temporal sensitivity, likely due to shorter processing times (Vatakis et al., 2012). With our stimulus selection, we thus aimed to represent audiovisual events of different dynamics.

Another question addressed in this study concerns the potential influence of available spatial details on audiovisual temporal integration. By adding Gaussian blur to the video sequences, we filtered out the finer visual details, but maintained the coarser outlines of the scenes (Thomas and Jordan, 2002). We predicted two possible outcomes of this manipulation, either an increased or a decreased perceptual sensitivity to audiovisual asynchrony. On the one hand, a discrepancy in quality between the audio and the video could separate the modalities in a domain additional to the temporal one. According to unitary theories on multisensory integration, the bond between modalities is strengthened by the characteristics they share (Welch and Warren, 1980; Vatakis and Spence, 2010). Hence, this divergence in signal quality could contribute to weaken the bond, possibly enhancing the effect of audiovisual asynchrony on the subjective temporal integration. On the other hand, the added distortion could mask the visual dynamics, making it harder to discern the temporal events for this modality. In turn, the temporal misalignment could become more difficult to make out, and audiovisual asynchrony could be tolerated at larger offsets. We explore these possibilites for the long and natural audiovisual content, as well as for the short and more isolated stimulus versions.

## 2. Materials and methods

We conducted a simultaneity judgement experiment to compare audiovisual temporal integration for short and isolated events and for longer and more eventful sequences. Importantly, the stimuli were not designed for research purposes. Instead, we selected our sequences from popular media outlets, aiming to include audiovisual content that represent what is typically encountered on TV or online. In so doing, we had no control over the scene compositions, any concurrent happenings, or the rate and extent of inherent motion. However, we did make sure that the events of interest were clearly visible within the shots and we avoided any abrupt starting or ending points. According to our aim to provide participants with more naturalistic multimedia experiences, we considered the long stimulus versions appropriate approximations. Yet, the combined durations neccessitated an adjustment of the full length of the experiment. Consequently, we only included two repetitions of each stimulus condition, and we ran the experiment in two separate sessions.

### 2.1. Participants

We ran the experiment with 19 native Norwegian participants (5 male, 14 female) aged between 19 and 41 years (*M* = 22.63, *SD* = 4.79). All participants reported normal hearing and normal or corrected vision. The study was conducted in accordance with national and international ethical standards, with participants providing consent prior to commencing the experiment. Additionally, we sought approval from the Norwegian Social Science Data Services to carry out our data collection.

### 2.2. Stimuli and material

**Figure 1:**
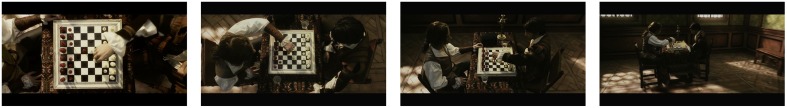
**The four screenshots represent the timeline of the long-running chess sequence; the first frame also illustrates the single event contained within the 1-s stimulus version**.

**Figure 2:**
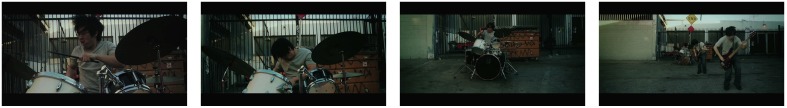
**The four screenshots represent the timeline of the long-running drums sequence; the first frame also illustrates the single event contained within the 1-s stimulus version**.

**Figure 3:**
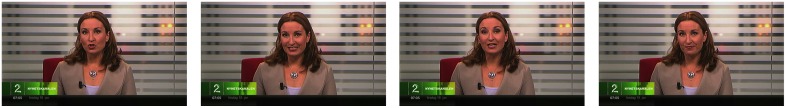
**The four screenshots represent the timeline of the long-running speech sequence**.

**Figure 4:**
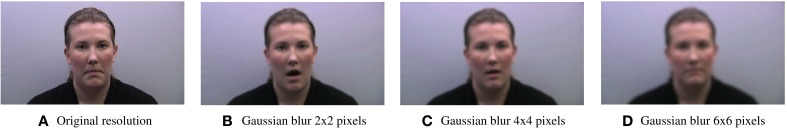
**Screenshots of single frames included in the 1-s presentations of the /BA/ syllable, at the original 1024×576 pixel resolution **(A)**, with Gaussian blur at 2×2 pixels **(B)**, Gaussian blur at 4×4 pixels **(C)**, and Gaussian blur at 6×6 pixels **(D)****.

The long stimuli (13 s) include a game of chess from a movie set in the Renaissance (Figure 1), a young man playing the drums (Figure 2), and a female news anchor filmed in a studio (Figure 3). For the chess and drums content, we derived short stimuli (1 s). by selecting a single event from each sequence, whereas the short speech stimulus used a spoken syllable from a separate recording (Figure 4). Additional details on the content and scene compositions are outlined in Table 1.

**Table 1:** **Detailed descriptions of the long and short audiovisual sequences presented to participants**.

**Chess content**	**Drums content**	**Speech content**
The video portrays a game of chess played by two young men in a Renaissance setting. In the opening scene of the long version, the chessboard and the players' hands are seen from above. The camera slowly zooms out and pans down to gradually include the two players and the surrounding room. Five pieces are picked up, moved and put down during the 13 s presentation. The short sequence includes a single chess move, from an overhead view. The content was sampled from the movie Assassin's Creed: Lineage (Part 1), with permission from Ubisoft.	A young man introduces the 13-s music sequence by hitting his drumsticks together three times, using wide and rapid, but visible, movements. He then commences to play the drums, while the camera zooms slowly out to include the alley where he sits. The sequence concludes with the appearance of first one, then two, of the drummer's clones, both with bass-guitars. In the 1-s excerpt, only one instance of the drumsticks hitting together is presented. The video was produced by Freddie Wong and Brandon Laatsch for the freddiew channel on YouTube.	The long sequence shows a female news anchor in studio, she presents a story about the return of two injured football. (Norwegian transcript: *Så sport. Flere friskmeldte ringrever tilbake på parketten, så gikk det Robert Hedins og Norges vei i går kveld. Seieren over Ø*sterrike betyr at alle muligheter er åpne.) The broadcast comes with permission to use for research purposes from the National Library of Norway. The short version presents a different female, speaking the single syllable /ba/. This recording comes from the Speech Lab at NTNU.

To keep the conditions that could be controlled as equal as possible, the average audio intensity was set to 70 dB and resolution to 1024 × 576 pixels. We implemented audiovisual asynchrony by adjusting the audio tracks in Audacity version 2.0.1 and editing out the selected asynchrony duration either at the beginning or at the end of the track. These durations corresponded to the lead or lag-time, so that the sound would start playing slightly sooner or slightly later with respect to the video track. The edited audio files were imported in the audio interchange file format (AIFF) to Final Cut Pro X and exported with the video track using the accompanying Compressor software and the H.264 encoder to convert files to QuickTime movies. Fade-ins and fade-outs were used to avoid giving away temporal cues; video onsets and offsets remained the same throughout for the same reason.

Initially, we selected temporal offsets from prior research (Conrey and Pisoni, 2006; Vatakis and Spence, 2006; van Wassenhove et al., 2007; Vroomen and Stekelenburg, 2011) and ran a pilot study with asynchrony levels increasing by 50 ms up to 500 ms audiovisual temporal separation. Results from the pilot demonstrated the characteristic asymmetry in sensitivity to audiovisual asynchrony, with audio lead detected at shorter offsets than audio lag asynchrony. Thus, the final selection of asynchrony levels were based on the distribution of synchrony responses and included the objectively synchronous condition, as well as temporal offsets of 50, 100, 150, and 200 ms, for audio lead, and 100, 200, 300, and 400 ms, for audio lag. Importantly, the technology used to assess the perception of synchrony can itself introduce asynchrony. Misalignments of the audio and video tracks are almost unavoidable when the streams are encoded and compressed separately; however, the introduced variations can be controlled in retrospect with the correct equipment (Maier et al., 2011). We did the synchrony controls manually, by going through the exported video files frame by frame and cross-referencing visual events with the spectrograms of their auditory counterparts.

To investigate the importance of available spatial details, we also applied Gaussian blur to the experiment videos at four different levels of distortion. Gaussian blur was chosen to distort the visual signal due to its ability to filter out high spatial frequencies and fine details while preserving global outlines. Following earlier work on degraded visual stimuli (MacDonald et al., 2000; Thomas and Jordan, 2002; Eg and Behne, 2009) and another pilot, blur filters were applied at three levels, 2 × 2, 4 × 4, and 6 × 6 pixels, in addition to the undistorted condition. Examples of the distortion levels, applied to the syllable stimulus, are presented in Figure 4.

With three content types, two sequence durations, nine temporal alignments, and four blur distortion levels, the full set of stimuli came to 216. Due to the accumulated time required to present all factorial levels for the fairly long stimuli, we limited the experiment to two repetitions and a total of 432 experiment trials. Videos were presented using Superlab 4.5 running on iMac 7.1 computers with 24 inch monitor resolution of 1920 × 1200 pixels, with video presentations sized to 1440 × 810 pixels. Audio was presented through AKG K271 circumaural headphones and responses were recorded by Cedrus RB-530 response pads.

### 2.3. Procedure

The experiment was conducted in the Speech Lab at the Norwegian University of Science of Technology. Although participants were free to move their seats, we encouraged them to find a comfortable position close to the monitor. Their task was to evaluate the synchrony of audiovisual presentations, indicating if the stimuli were synchronous or asynchronous. The experiment ran over two sessions, set 1 week apart. Each session included one repetition of every stimulus, randomized separately for all participants and between the individual sessions. At the beginning of the first session, participants were requested to read through an information sheet, give written consent, and provide some background information. They then received instructions on how the experiment would progress and how to handle the headphones and the response pad. The latter included two labeled buttons, *SYNC* for presentations perceived to be synchronous, and *ASYNC* for presentations perceived to be asynchronous. Two practice trials introduced the experiment, and on these participants received feedback on their response accuracy. A break was included halfway through the experiment sessions. Participants progressed at their own pace, thus the total duration of each session varied between 50 and 70 min.

## 3. Results

**Figure 5:**
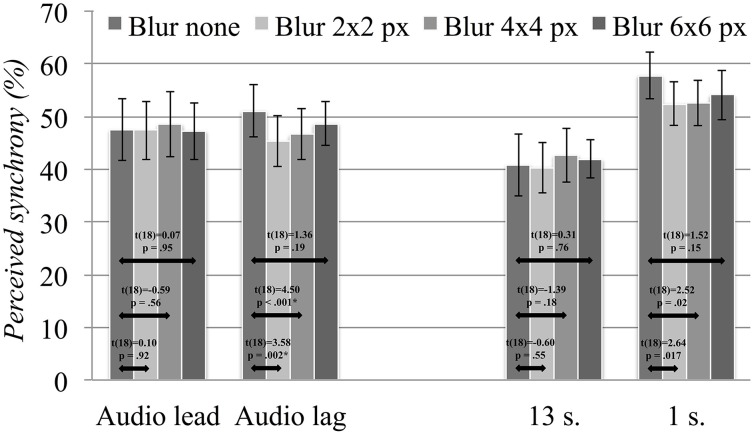
**Overall rates of perceived synchrony averaged across all asynchronous presentations, separated by direction of asynchrony (left side) and stimulus duration (right side)**. Error bars represent the 95% confidence intervals and results from the *post-hoc* paired-comparison *t*-tests are overlaid, with asterisks marking significant contrasts.

Initially, we decided to explore the impact of video blur on the rate of reported synchrony for asynchronous presentations. Considering the different scales applied to audio lead and audio lag asynchrony levels, along with the inclusion of only two stimulus repetitions, we ran the first analysis with asynchrony direction as a separate factor and averaged across the respective asynchrony levels. The results from the four-way repeated measures ANOVA are summarized in Table 2. While we found no main effect for blur, the analysis revealed significant interactions with duration and with asynchrony direction. Figure 5 presents our *post-hoc* explorations of these interactions, using paired comparison *t*-tests and applying Holm's sequential Bonferroni adjustments (Holm, 1979) (significance criteria for six comparisons: 0.008 for smallest significant *p*-value, 0.01 for the second smallest, 0.013 for the third, 0.017 for the fourth, 0.025 for the fifth, and 0.05 for the sixth). We found some significant variations across blur levels for audio lag presentations; specifically, we found small reductions in reported synchrony going from the full quality videos to the mid-levels of blur distortion. However, the reductions in reported synchrony were fairly small, with the largest drop observed between the undistorted condition and the least distorted of the blur conditions. For these, the rate of reported synchrony increased again as the visual distortions became more severe. Alongside their small effect sizes, we found no clear indications that the blur manipulation influenced the rate of perceived audiovisual synchrony.

**Table 2:** **ANOVA results for asynchrony direction, stimulus duration, content and blur levels, significant results are marked with asterisks**.

**Factors**	**ANOVA statistics**
Blur	*F*_(3, 54)_ = 1.63, *P* = 0.19, η^2^_p_ = 0.08
Duration	*F*_(1, 18)_ = 84.22, *p* < 0.001^*^, η^2^_p_ = 0.82
Content	*F*_(2, 36)_ = 8.31, *P* = 0.001^*^, η^2^_p_ = 0.32
Asynchrony direction	*F*_(1, 18)_ = 0.01, *P* = 0.92, η^2^_p_ = 0.00
Blur^*^Duration	*F*_(3, 54)_ = 3.14, *P* = 0.03^*^, η^2^_p_ = 0.15
Blur^*^Content	*F*_(6, 108)_ = 1.08, *P* = 0.38, η^2^_p_ = 0.06
Blur^*^Asynchrony direction	*F*_(3, 54)_ = 2.98, *P* = 0.04^*^, η^2^_p_ = 0.14
Duration^*^Content	*F*_(2, 36)_ = 6.68, *P* = 0.003^*^, η^2^_p_ = 0.27
Duration^*^Asynchrony direction	*F*_(1, 18)_ = 15.16, *P* = 0.001^*^, η^2^_p_ = 0.46
Content^*^Asynchrony direction	*F*_(2, 36)_ = 97.41, *p* < 0.001^*^, η^2^_p_ = 0.84
Blur^*^Duration^*^Content	*F*_(6, 108)_ = 2.64, *P* = 0.02^*^, η^2^_p_ = 0.13
Blur^*^Duration^*^Asynchrony direction	*F*_(3, 54)_ = 0.09, *P* = 0.97, η^2^_p_ = 0.01
Blur^*^Content^*^Asynchrony direction	*F*_(6, 108)_ = 1.74, *P* = 0.12, η^2^_p_ = 0.09
Duration^*^Content^*^Asynchrony direction	*F*_(2, 36)_ = 1.17, *P* = 0.32, η^2^_p_ = 0.06
Blur^*^Duration^*^Content^*^Async direction	*F*_(6, 108)_ = 1.20, *P* = 0.31, η^2^_p_ = 0.06

Consequently, we set out to investigate the collected data without blur as a separate factor. We ran the next statistical analyses separately for the two types of asynchrony and included blur distortion levels as repetitions. This resulted in two repeated-measures ANOVAs into the effects of stimulus duration, content type, asynchrony, and their interactions, on audiovisual temporal integration. Results from the full analyses are summarized in Table 3 and they suggested that these manipulations did influence participants' perception of synchrony. Moreover, the effect sizes, calculated as partial eta-squared (η^2^_p_), showed that content type and duration contributed to fairly large changes in perceived synchrony in the audio lead direction.

**Table 3:** **Results from ANOVAs exploring content and duration for audio lead and audio lag conditions, significant results are marked with asterisks**.

	**Factors**	**ANOVA statistics**
Audio lead	Duration	*F*_(1, 18)_ = 64.54, *p* < 0.001^*^, η^2^_p_ = 0.78
	Content	*F*_(2, 36)_ = 60.51, *p*<0.001^*^, η^2^_p_ = 0.77
	Asynchrony	F_(4, 72)_ = 234.38, p<0.001^*^, η^2^_p_ = 0.93
	Duration^*^Content	*F*_(2, 36)_ = 4.45, *p* = 0.02^*^, η^2^_p_ = 0.20
	Duration^*^Asynchrony	*F*_(4, 72)_ = 15.66, *p*<0.001^*^, η^2^_p_ = 0.47
	Content^*^Asynchrony	*F*_(8, 144)_ = 14.86, *p*<0.001^*^, η^2^_p_ = 0.45
	Duration^*^Content^*^Asynchrony	*F*_(8, 144)_ = 5.47, *p*<0.001^*^, η^2^_p_ = 0.23
Audio lag	Duration	*F*_(1, 18)_ = 5.18, *p* = 0.04^*^, η^2^_p_ = 0.22
	Content	*F*_(2, 36)_ = 16.60, *p*<0.001^*^, η^2^_p_ = 0.48
	Asynchrony	*F*_(4, 72)_ = 343.59, *p*<0.001^*^, η^2^_p_ = 0.95
	Duration^*^Content	*F*_(2, 36)_ = 5.33, *p* = 0.01^*^, η^2^_p_ = 0.23
	Duration^*^Asynchrony	*F*_(4, 72)_ = 10.67, *p*<0.001^*^, η^2^_p_ = 0.37
	Content^*^Asynchrony	*F*_(8, 144)_ = 10.84, *p*<0.001^*^, η^2^_p_ = 0.38
	Duration^*^Content^*^Asynchrony	*F*_(8, 144)_ = 3.52, *p* = 0.001^*^, η^2^_*p*_ = 0.16

**Figure 6:**
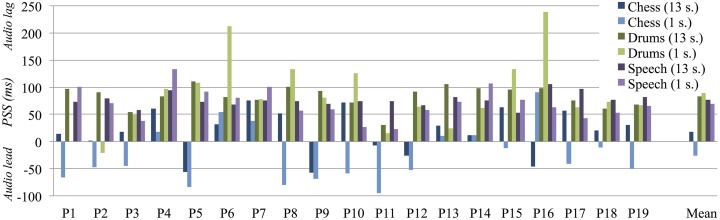
**Points of subjective simultaneity (PSS) for all stimulus contents and durations, plotted for each participant as well as the overall mean**. The PSS values spread across a large range of audiovisual asynchrony from ≈100 ms audio lead to ≈250 ms audio lag.

We then performed individual Gaussian curve fittings for every participant's responses to each stimulus type. We assessed the goodness of fit of the individual Gaussian curves using both the coefficient of determination, *R*^2^, and the Shapiro-Wilk test of normality, presented in Table 4. The first yields score that denotes the distance between each data-point and the fitted curve, whereas the second is a measure of how well the curve conforms to a normal distribution (Razali and Wah, 2011). From the curve fittings we derived the full width at half maximum (FWHM); this measure of the curve's width at the point where a participant is equally likely to report synchrony as asynchrony represents the individual's window of temporal integration (Conrey and Pisoni, 2006). The means of the Gaussian curves define the points of subjective simultaneity (PSS). Considering that subjective synchrony rarely corresponds to the objective synchrony, the PSS provides a measure of the optimal degree of asynchrony for each individual's perception of temporal coherence. As the term indicates, these mean points are highly subjective and we observed large variations across participants, visualized in the plot in Figure 6.

**Table 4:** **Goodness of fit of individual Gaussian curves, with the lowest, highest, average and standard deviation (*****SD*****) of participants' scores, represented by the coefficient of determination**, ***R*****^2^, and the Shapiro-Wilk test of normality**.

	***R*^2^**			**Shapiro-wilk**		
	**Min**.	**Max**.	**Mean (*SD*)**	**Min**.	**Max**.	**Mean (*SD*)**
Chess (13 s)	0.79	0.98	0.89 (0.05)	0.87	0.98	0.93 (0.03)
Chess (1 s)	0.74	0.98	0.89 (0.07)	0.89	0.98	0.93 (0.03)
Drums (13 s)	0.61	0.94	0.85 (0.07)	0.75	0.97	0.92 (0.05)
Drums (1 s)	0.60	0.95	0.82 (0.12)	0.76	0.97	0.91 (0.06)
Speech (13 s)	0.78	0.97	0.88 (0.05)	0.88	0.97	0.93 (0.03)
Speech (1 s)	0.70	0.95	0.83 (0.06)	0.87	0.99	0.94 (0.04)

**Figure 7:**
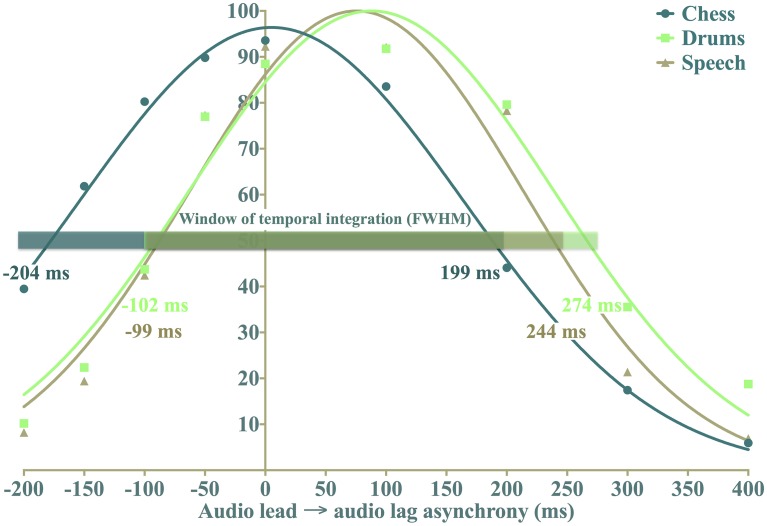
**Perceived synchrony distributions for each content type, fitted to the displayed mean points that correspond to the average of all participants' means for the presented asynchrony and content conditions**. The overlaid windows of temporal integration are calculated from the mean FWHM values, resulting in some discrepancy between the derived audio lead and lag thresholds and the visual curve examples.

By subtracting the PSS from half the FWHM, we calculated audio lead thresholds; similarly, we obtained audio lag thresholds by adding the PSS to half of the FWHM. The windows of temporal integration and the asynchrony detection thresholds are portrayed in Figure 7. Prior to further analysis, we performed an outlier detection and found that one participant's PSS for the short drums content fell outside the presented range of asynchrony (556 ms audio lag), with a FWHM value almost three times greater than the average (1241 vs. 425 ms). Consequently, we excluded the participant's responses for this content.

Running a repeated-measures ANOVA for FWHM uncovered significant results for the main effects of stimulus duration, *F*_(1,17)_ = 39.19, *p* < 0.001, η^2^_p_ = 0.70, and content, *F*_(2,34)_ = 3.59, *P* = 0.04, η^2^_p_ = 0.17, but no significant interaction between the two, *F*_(2,34)_ = 0.67, *P* = 0.52. These findings point to a wider window of temporal integration for short (*M* = 432 ms) vs. long (*M* = 317 ms) stimulus duration, they also suggest that the window of temporal integration depends on the nature of the audiovisual presentation. The latter notion was explored further with three *post-hoc* paired comparison *t*-tests with Holm's sequential Bonferroni adjustments (0.017 for smallest significant *p*-value, 0.025 for the second-smallest, and 0.05 for the third), the results of which are listed in Table 5. Although the *post-hoc* comparisons found no signficant differences between content types, the window of temporal integration is 60 ms wider for chess than for speech, as seen in Figure 7. Combined with the main effect that indicates variations in perceived synchrony across the three contents, these findings do hint at a tendency to detect asynchrony at shorter temporal offsets with asynchronous speech, compared to asynchronous drumming.

**Table 5:** **Summary of paired comparison** ***t*****-tests for FWHM and PSS, significant results are marked with asterisks**.

	**Paired comparison**	***T*-test statistics**
FWHM	Chess vs. Drums	*t*_(17)_ = 1.29, *P* = 0.21
	Chess vs. Speech	*t*_(18)_ = 2.24, *P* = 0.04
	Drums vs. Speech	*t*_(17)_ = 1.89, *P* = 0.08
PSS	Chess vs. Drums	*t*_(17)_ = −8.97, *p* < 0.001^*^
	Chess vs. Speech	*t*_(18)_ = −10.27, *p* < 0.001^*^
	Drums vs. Speech	*t*_(17)_ = 1.64, *P* = 0.12
	Chess (13 s) vs. Chess (1 s)	*t*_(18)_ = 3.18, *P* = 0.005^*^
	Drums (13 s) vs. Drums (1 s)	*t*_(17)_ = −0.45, *P* = 0.66
	Speech (13 s) vs. Speech (1 s)	*t*_(18)_ = 1.03, *P* = 0.32

Another repeated-measures ANOVA, run for PSS, revealed a main effect of content, *F*_(2,34)_ = 60.76, *p* < 0.001, η^2^_p_ = 0.78), but none for stimulus duration, *F*_(1,17)_ = 3.10, *P* = 0.10, although their interaction yielded a significant result, *F*_(2,34)_ = 4.82, *P* = 0.01, η^2^_p_ = 0.22). While the main effect for content implies a shift in the subjective sensitivity to temporal misalignment, dependent on the nature of the audiovisual event, the interaction with duration indicates that this sensitivity also varies with the number of presented events. We followed up with *post-hoc* paired comparison *t*-tests to assess differences across the specific content types and stimulus durations, again with Holm-Bonferroni corrections for three contrasts; the results are presented in Table 5. While we uncovered no differences between drums and speech, the PSS scores fell significantly closer to objective synchrony for the chess sequences, compared to the other two content types. Furthermore, only the chess content revealed a significant difference between the 13 and 1 s durations, despite the low PSS values. Figure 6 demonstrates how the subjective mean point corresponds to audio lag asynchrony for the longer duration and to audio lead for the shorter duration.

**Figure 8:**
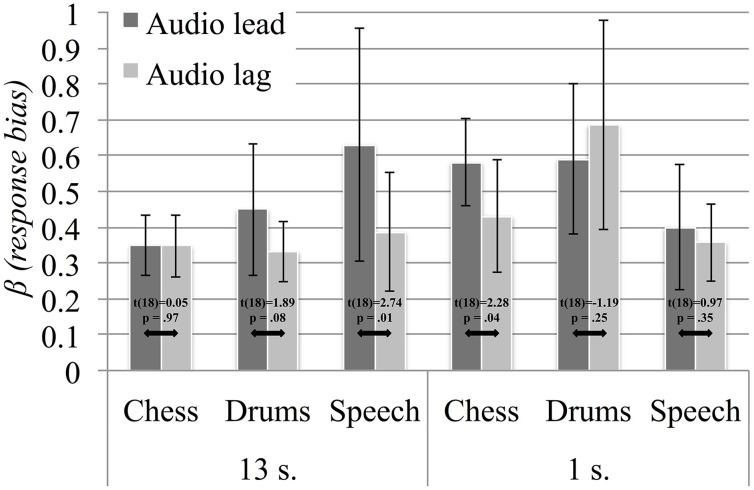
**Response bias β measures for long and short chess, drums and speech sequences, averaged across participants**. Error bars represent the 95% confidence intervals, no significant contrasts where found with the Holm-Bonferroni adjustments.

**Figure 9:**
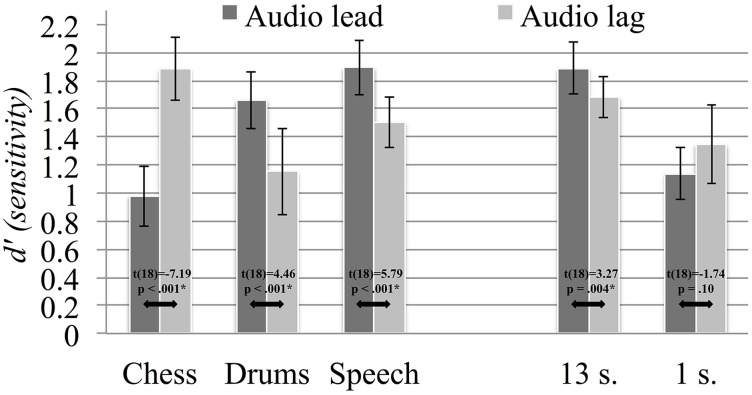
**Signal detection d' sensitivity values, grouped according to content type and stimulus duration, averaged across participants**. Error bars represent the 95% confidence intervals and asterisks denote significant contrasts.

We ran an additional signal detection analysis in order to evaluate whether the detection and sensitivity to audiovisual asynchrony depended on the precedence of the auditory and visual signals. Following the definitions described in Kelly and Matthews (2011), we considered a “hit” as a correctly identified asynchronous trial and a “miss” as the failure to detect an asynchronous trial. We relied on the procedures of Stanislaw and Todorov (1999) to calculate β and d' scores for lead and lag asynchrony trials (averaged across asynchrony levels). Following these, we obtained measures of the participants' β response bias, reflecting their tendencies to provide either a *SYNC* or an *ASYNC* response to the two types of asynchrony. Moreover, the d' sensitivity scores reflect participants' abilities to correctly identify asynchronous trials for audio lead and audio lag asynchrony. We ran two repeated measures ANOVAs to assess how the scores varied between lead and lag asynchrony, as well as between the two durations and the three content types. The results summary in Table 6 show a main effect of asynchrony direction only for β, pointing to a higher response bias for audio lead (*M* = 0.50), compared to audio lag (*M* = 0.42), asynchrony. We followed up the significant three-way β interaction by carrying out *post-hoc* paired comparison *t*-tests with Holm-Bonferroni adjustments, with results presented visually in Figure 8. As seen, response bias did not differ significantly between audio lead and audio lag asynchrony for long nor for short stimuli. Overall, we observed a greater propensity to provide *ASYNC* responses when the auditory signal preceded the visual signal; however, this trend was not consistent, particularly not for the 1 s sequences. The analysis of d' scores revealed main effects of both duration (*M*_13 *s*_ = 1.78, *M*_1 *s*_ = 1.24) and content (*M*_chess_ = 1.42, *M*_drums_ = 1.41, *M*_speech_ = 1.70), and significant interactions with asynchrony direction for both factors. In other words, asynchrony was correctly identified at a higher rate for long than for short stimuli, and for speech compared to chess and drums stimuli. We explored the interactions with *post-hoc* paired-comparison *t*-tests, which are presented in Figure 9. Judging by the inconsistencies in the significant differences in sensitivity scores between audio lead and audio lag asynchrony across content types and stimulus durations, our participants did not exhibit a systematic sensitivity to either type of asynchrony. Still, the shift in the chess stimuli's PSS measures toward objective synchrony could be related to the seeming difficulty in correctly identifying audio lead asynchrony for this content. In contrast, participants displayed greater sensitivity to audio lag asynchrony for the drums and speech stimuli, suggesting that the temporal perception of the two rely on more similar mechanisms.

**Table 6:** **Results from ANOVAs run separately for d' (sensitivity) and β (response bias), significant results are marked with asterisks**.

	**Factors**	**nANOVA statistics**
β	Asynchrony direction	*F*_(1, 18)_ = 7.34, *P* = 0.01^*^, η^2^_p_ = 0.29
	Duration	*F*_(1, 18)_ = 2.00, *P* = 0.18, η^2^_p_ = 0.10
	Content	*F*_(2, 36)_ = 0.70, *P* = 0.50, η^2^_p_ = 0.04
	Asynchrony direction^*^Duration	*F*_(1, 18)_ = 2.00, *P* = 0.17, η^2^_p_ = 0.10
	Asynchrony direction^*^Content	*F*_(2, 36)_ = 2.04, *P* = 0.15, η^2^_p_ = 0.10
	Duration^*^Content	*F*_(2, 36)_ = 4.73, *P* = 0.02^*^, η^2^_p_ = 0.21
	Asynchrony direction^*^Duration^*^Content	*F*_(2, 36)_ = 7.17, *P* = 0.002^*^, η^2^_p_ = 0.29
d'	Asynchrony direction	*F*_(1, 18)_ = 0.001, *P* = 0.98, η^2^_p_ = 0.00
	Duration	*F*_(1, 18)_ = 25.85, *p*<0.001^*^, η^2^_p_ = 0.59
	Content	*F*_(2, 36)_ = 3.99, *P* = 0.03^*^, η^2^_p_ = 0.18
	Asynchrony direction^*^Duration	*F*_(1, 18)_ = 18.50, *p*<0.001^*^, η^2^_p_ = 0.51
	Asynchrony direction^*^Content	*F*_(2, 36)_ = 99.34, *p*<0.001^*^, η^2^_p_ = 0.85
	Duration^*^Content	*F*_(2, 36)_ = 1.41, *P* = 0.26, η^2^_p_ = 0.07
	Asynchrony direction^*^Duration^*^Content	*F*_(2, 36)_ = 1.86, *P* = 0.17, η^2^_p_ = 0.09

## 4. Discussion

This study was motivated by the notion that the perception of synchrony could be connected to the dynamics and number of corresponding audiovisual events, among other variables. Considering the large number of studies that rely on simple and isolated audiovisual presentations in the exploration of their temporal integration, we aimed to extend our investigation to content that are more representative of what we meet in everyday life. To explore perceived synchrony across content types, we sampled audiovisual material from typical multimedia outlets. We retrieved three sequences, a chess game, a drum solo, and a news broadcast, to represent action, music, and speech content, respectively. Furthermore, we also set out to explore the subjective ability to discern and align the auditory and visual modalities for events of different dynamics. In this pursuit, we isolated a single audiovisual event for each content type, enabling a comparison between short and controlled stimuli with long-running stimuli that contained continuous and repeated movements. Keeping in mind that we presented participants with excerpts from broadcasts originally created for entertainment purposes, we consider this work a contribution to the small research field that apply more ecologically valid and representative stimuli to the study of multisensory perception. Although the derived windows of temporal integration showed variations across participants, content types and stimulus durations, the collected data yielded typical distributions of perceived synchrony that correspond to earlier studies that have applied the same methodology (Conrey and Pisoni, 2006; Petrini et al., 2009; Stevenson and Wallace, 2013). Even though results from this line of experimentation is bound to be noisy, we would claim that research on audiovisual processes is ready to broaden its scope when it comes to stimulus material. Combined with well-established theories on human multisensory perception and findings from lower-level perceptual processes, this type of study can shed further light on human multisensory perception in the real world.

In line with predictions, our analyses showed that the perception of synchrony for the longer and more dynamic audiovisual events is more sensitive to temporal misalignments than for the short and isolated events. This trend was apparent from the wider windows of temporal integration for all short stimuli, compared to their longer counterparts. Because the isolated chess and drums stimuli are excerpts from the 13-s versions, the difference between them is unlikely to arise from possible distractors contained in the scenes. Instead, the greater perceptual tolerance to asynchrony for the simple stimuli may be attributed to the isolation of the events within them. With only one temporal event presented during the 1-s presentations, participants may find it more difficult to judge the simultaneity of the auditory and the visual events. In contrast, the repeated nature of the long sequences provides participants with multiple reference points, facilitating a more accurate temporal alignment of the auditory and visual signals. Accordingly, the use of isolated events could contribute to overestimating the temporal tolerance of the human perceptual system, whereas a dynamic sequence of events, similar to so many natural actions, may serve as a more accurate representation of real-life perception.

That said, this rationale can seem inconsistent with the findings of Vatakis and Spence (2006), where participants judged temporal order more accurately for action stimuli than for music or speech stimuli. In comparison to their action sequences that contained a single audiovisual event, their music and speech stimuli were continuous in nature. Yet, unlike our manipulation for the short stimulus duration, their action events were dynamic, presumably preserving the full extent of the hitting movements. With these types of movement follow highly predictable moments of impact that could serve as salient temporal cues, allowing for more accurate temporal judgements. Thus, variations in perceived synchrony between action, music and speech could just as well relate to the movements, visual angles, sound onsets and offsets, along with numerous other distinctive features inherent in the events. Moreover, our comparisons of content types show the same difference between speech and action-oriented sequences that have been presented by others (Dixon and Spitz, 1980; Vatakis and Spence, 2006; Stevenson and Wallace, 2013), with audiovisual asynchrony detected at shorter intervals for the more predictable action events.

In the exploration of differences between audiovisual events, we also found that the subjective perception of synchrony remained fairly consistent across the speech and drum contents, but shifted significantly in the audio lead direction for the chess content. Most of our participants perceived the short chess sequence as synchronous only when the audio preceded the video. Thus, the temporal integration of the chess sequences is distinguishable from the other two events. Moreover, the short stimulus yielded PSS displaced from the more typical values derived from this methodology (Zampini et al., 2005; Conrey and Pisoni, 2006; van Eijk et al., 2008; Petrini et al., 2009; Vroomen and Stekelenburg, 2011), which arguably also adhere to the physical laws of nature. Although we can only speculate on the reason for this temporal displacement, we believe it could be related to the visual angle. Where the drummer and the two female speakers are filmed from the front, the chess sequence starts out with a bird's eye view of the game, before the camera zooms out and pans down to include the full scene with the two players. The similarity in perceived synchrony between the drums and speech content might be attributed to their dynamics and the numerous audiovisual events that take place in the 13 s sequences, which stand out from the five chess pieces that are moved in the chess sequence. However, the similarity between the drums and speech stimuli may also indicate that temporal integration does not depend on the nature of the event, but rather the predictability and visibility of the event. In the short chess version, the top-down shot included only the chessboard and the hands of the players. This visual angle could potentially make it difficult to discern the precise moment of impact, particularly prior to the event; this in turn would give leeway for the early arrival of the corresponding sound. As mentioned, these are only speculations, but we hope to see future investigations into the relative ease of making temporal judgements for impact actions observed from different viewpoints.

As others have noted before us (Lewkowicz, 1996; Grant et al., 2003), the perception of audiovisual synchrony is not identical for auditory and visual signal precedence. Audio lead asynchrony tends to be detected at shorter temporal offsets than audio lag asynchrony, which was also evident for our results. To explore this phenomenon more closely, we carried out signal detection analyses and compared response bias and sensitivity scores for the two asynchrony directions, along with stimuli content and duration. While we found that participants were better at detecting asynchrony when the auditory signal arrived before the visual, this trend was not consistent. In line with the described skew in perceived synchrony between chess and the other two contents, the sensitivity scores were higher for audio lead for both drums and speech, but not for chess. These results put further emphasis on the distinction of the chess content.

With respect to the loss of visual details, we found only small variations in temporal integration across our blur manipulations. In our earlier work (Eg et al., 2015), we carried out a comprehensive investigation of perceived synchrony for distorted audiovisual content, concluding that the temporal integration of the two modalities is fairly robust to the loss of sensory information. Here we observe that this robustness holds true for both short and long-running stimuli. We again surmise that fine-grained spatial details are not required in order for the perceptual system to align the two modalities in time.

In addition to differences in perceived synchrony observed across stimulus content and duration, our results showed great variations across participants. This variance was evident both with respect to the temporal tolerance to asynchrony and for the peak in subjective synchrony, with participant averages that were separated by more than 200 ms. The full range of PSS scores is visualized in Figure 6. A large degree of variation is only to be expected when introducing less controlled experimental stimuli; it is therefore important to be aware of this possible outcome when designing a study. When including more complex and natural auditory or visual presentations, it seems prudent to increase the number of repetitions and participants. Of course, this may come at the expense of the number of manipulations to explore, a consideration that may decrease the motivation to pursue this line of investigation.

All in all, our findings speak in favor of introducing more natural stimuli in studies on audiovisual perception. Applying stimuli with either one or several audiovisual events allows for direct comparisons of the measure at hand, shedding more light on the generalisability of results. In turn, we may learn more about the human perceptual system, and how it processes the noisy signals that surround us in the real world.

### Conflict of interest statement

The authors declare that the research was conducted in the absence of any commercial or financial relationships that could be construed as a potential conflict of interest.
